# Supplementary material for: Myoferlin alleviates pressure overload-induced cardiac hypertrophy and dysfunction by inhibiting NLRP3-mediated pyroptosis
Source: PeerJ. 2024 Nov 13;12:e18499. doi: 10.7717/peerj.18499 (PMC11568814; doi:10.7717/peerj.18499)
Supplement: Supplemental Information 2 [file peerj-12-18499-s002.docx]

**Supplementary Figure 1**

**
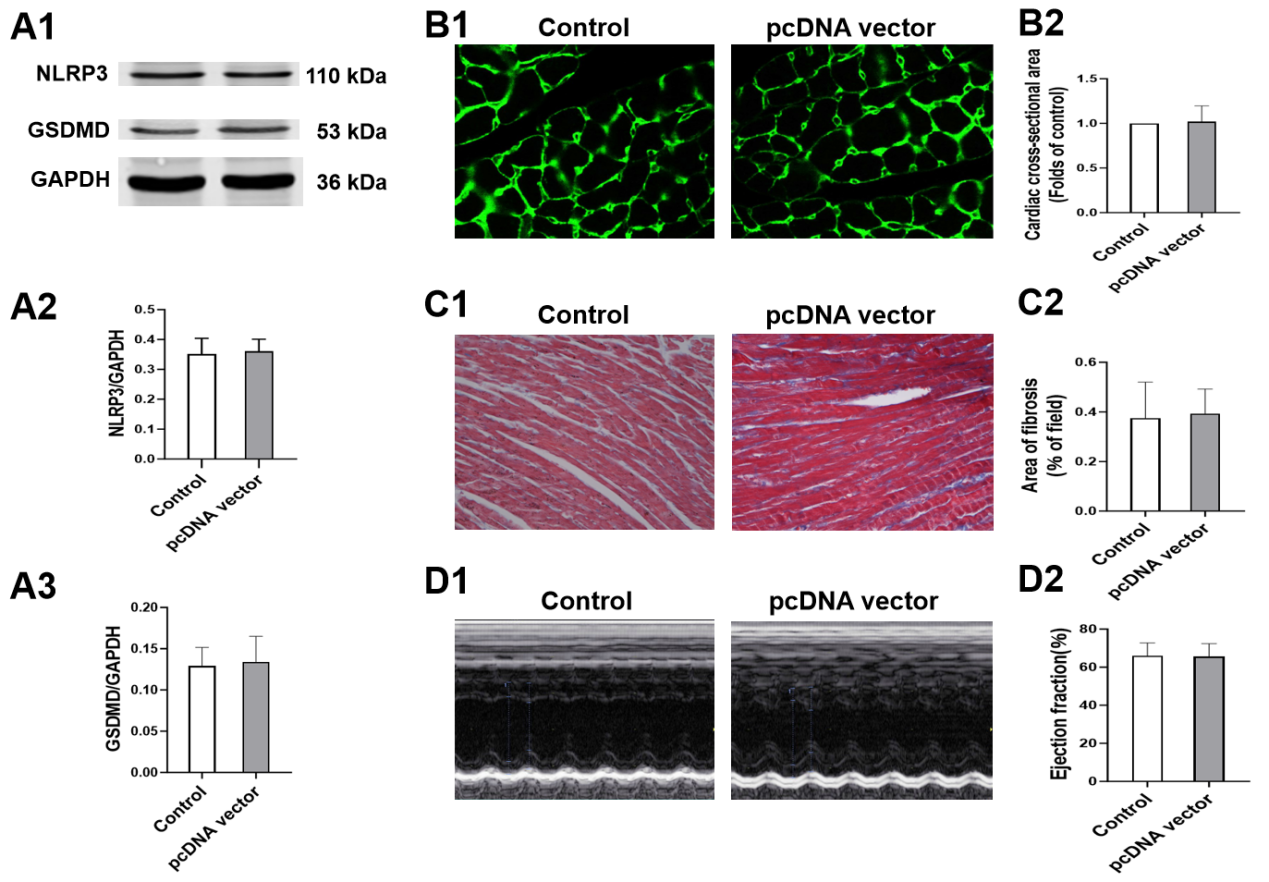
**

**Supplementary Figure 1.** Through the comparison of immunoblot-related proteins, echocardiography, myocardial fibrosis, and cross-sectional area of myocytes in both control mice and those treated with the pc-DNA vector, no significant differences were observed between the two groups, indicating that the pc-DNA vector has no impact on mice. A: Immunoblots showing protein levels of NLRP3( A2), GSDMD1(A3) in the suitably treated cardiomyocytes. B: Representative images and quantitative analysis of the cross-sectional area of cardiomyocytes in the control and pc-DNA vector group. C: Representative images and quantitative analysis of Masson's staining. D: Representative images of M-mode echocardiography and quantitative analysis of left ventricular ejection fraction (EF) in mice.

**Supplementary Figure 2**

**
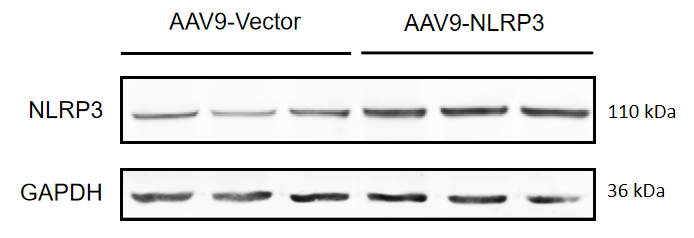
**

**Supplementary Figure 2.** Through immunoblot images, we demonstrated that the NLRP3 level in the AAV-NLRP3 group significantly increased, confirming the successful overexpression of NLRP3.
