# Supplementary figures and images for: Myoferlin alleviates pressure overload-induced cardiac hypertrophy and dysfunction by inhibiting NLRP3-mediated pyroptosis
Source: PeerJ. 2024 Nov 13;12:e18499. doi: 10.7717/peerj.18499 (PMC11568814; doi:10.7717/peerj.18499)

Figure 2

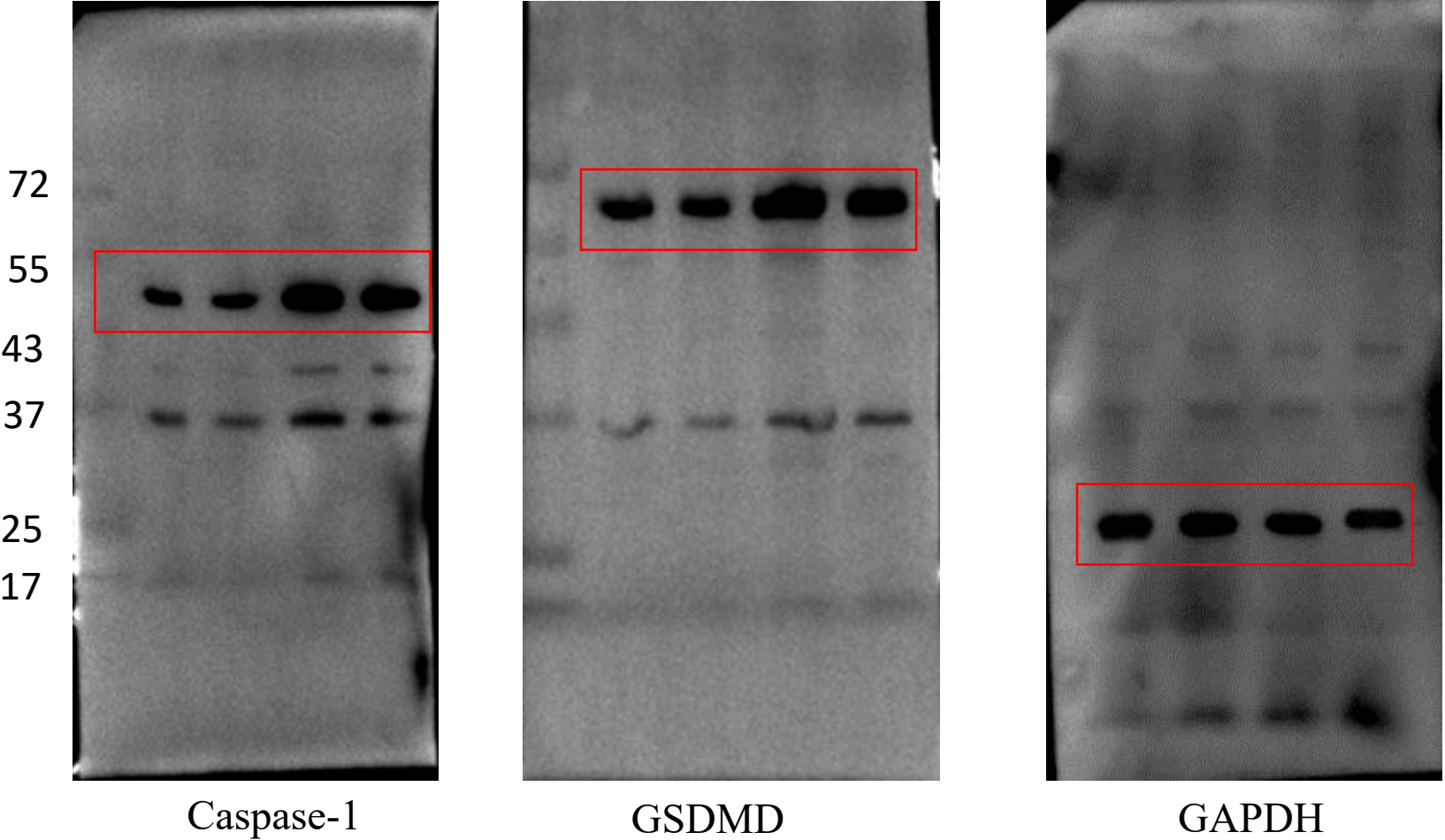

Figure 3

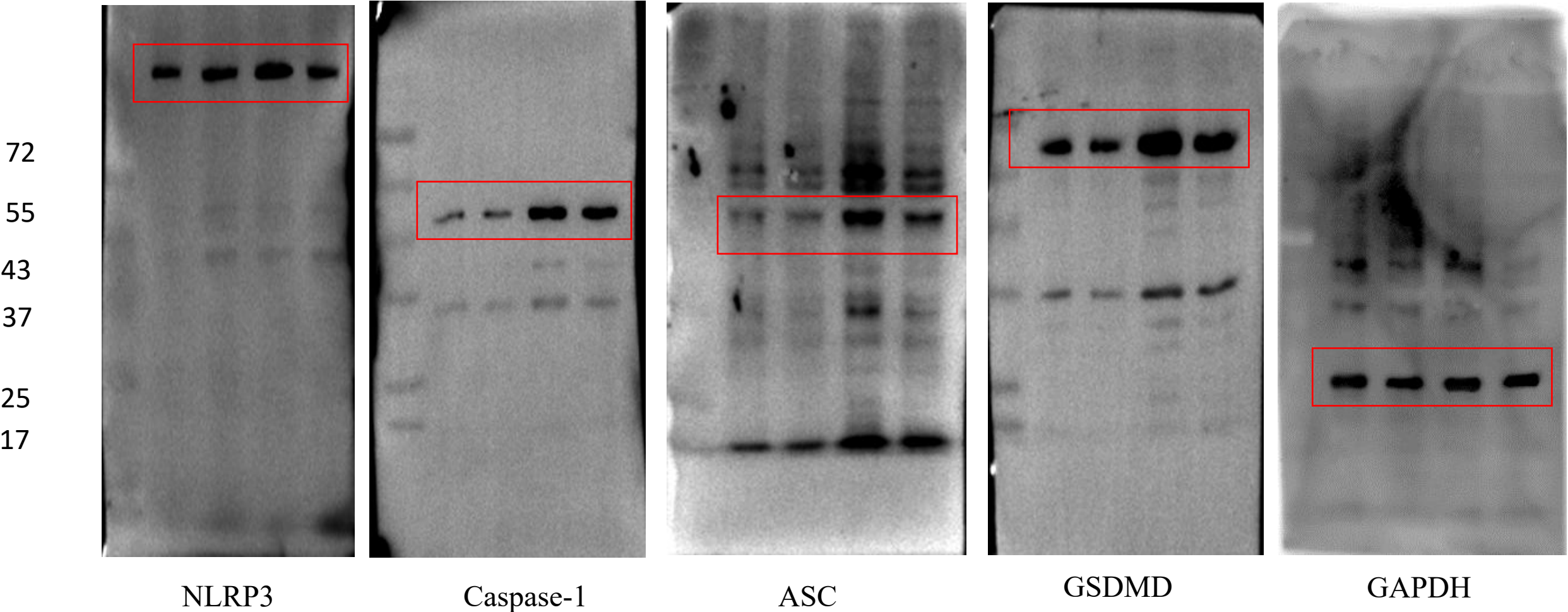

Figure 4

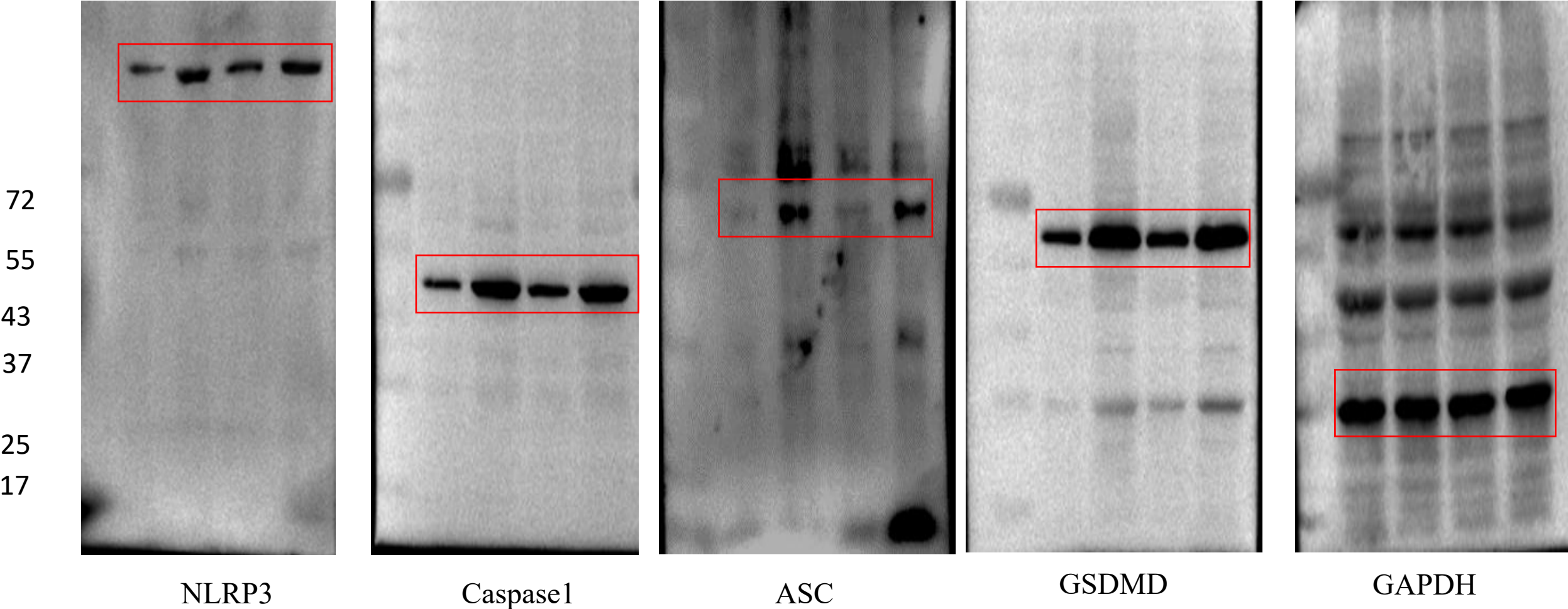

Supplement: Supplemental Information 3 [file peerj-12-18499-s003.pdf]
